# Acute kidney injury in critical ill patients affected by influenza A (H1N1) virus infection

**DOI:** 10.1186/cc10046

**Published:** 2011-02-22

**Authors:** Ignacio Martin-Loeches, Elisabeth Papiol, Alejandro Rodríguez, Emili Diaz, Rafael Zaragoza, Rosa María Granada, Lorenzo Socias, Juan Bonastre, Montserrat Valverdú, Juan Carlos Pozo, Pilar Luque, Jose Antonio Juliá-Narvaéz, Lourdes Cordero, Antonio Albaya, Daniel Serón, Jordi Rello

**Affiliations:** 1 Critical Care Department, Joan XXIII University Hospital-CIBER Enfermedades Respiratorias, URV, and IISPV, Mallafre i Guasch, ES-43007 Tarragona, Spain; 2 Critical Care Department, Hospital Dr. Peset, Gaspar Aguilar, ES-46017 Valencia, Spain; 3 Critical Care Department, Hospital de Bellvitge, Feixa Llarga, ES-08907 Barcelona, Spain; 4 Critical Care Department, Hospital Son Llatzer, Carretera Manacor, ES-07198 Mallorca, Spain; 5 Critical Care Department, Hospital La Fe, Avenida Campanar, ES-46009 Valencia, Spain; 6 Critical Care Department, Hospital Arnau, Av. Alcalde Rovira Roure, ES-25198 Lleida, Spain; 7 Critical Care Department, Hospital Reina Sofía, Avenida Menéndez Pidal, ES-14004 Córdoba, Spain; 8 Critical Care Department, Hospital Lozano Blesa, Avenida San Juan Bosco, ES-50009 Zaragoza, Spain; 9 Critical Care Department, Hospital Infanta Cristína, Avenida Huelva, 06005 ES-Badajoz, Spain; 10 Critical Care Department, CHUAC, Xubias de Arriba, ES-15006 A'Coruña, Spain; 11 Critical Care Department, Hospital de Guadalajara, C/Donante de Sangre, ES-19002 Guadalajara, Spain; 12 Nephrology Department Vall d'Hebron University Hospital, Passeig Vall d'Hebron, ES-08035 Barcelona, Spain; 13 Critical Care Department, Vall d'Hebron University Hospital, IRVH, CIBERes, Passeig Vall d'Hebron, ES-08035 Barcelona, Spain

## Abstract

**Introduction:**

Little information exists about the impact of acute kidney injury (AKI) in critically ill patients with the pandemic 2009 influenza A (H1N1) virus infection.

**Methods:**

We conducted a prospective, observational, multicenter study in 148 Spanish intensive care units (ICUs). Patients with chronic renal failure were excluded. AKI was defined according to Acute Kidney Injury Network (AKIN) criteria.

**Results:**

A total of 661 patients were analyzed. One hundred eighteen (17.7%) patients developed AKI; of these, 37 (31.4%) of the patients with AKI were classified as AKI I, 15 (12.7%) were classified as AKI II and 66 (55.9%) were classified as AKI III, among the latter of whom 50 (75.7%) required continuous renal replacement therapy. Patients with AKI had a higher Acute Physiology and Chronic Health Evaluation II score (19.2 ± 8.3 versus 12.6 ± 5.9; *P *< 0.001), a higher Sequential Organ Failure Assessment score (8.7 ± 4.2 versus 4.8 ± 2.9; *P *< 0.001), more need for mechanical ventilation (MV) (87.3% versus 56.2%; *P *< 0.01, odds ratio (OR) 5.3, 95% confidence interval (CI) 3.0 to 9.4), a greater incidence of shock (75.4% versus 38.3%; *P *< 0.01, OR 4.9, 95% CI, 3.1 to 7.7), a greater incidence of multiorgan dysfunction syndrome (92.4% versus 54.7%; *P *< 0.01, OR 10.0, 95% CI, 4.9 to 20.21) and a greater incidence of coinfection (23.7% versus 14.4%; *P *< 0.01, OR 1.8, 95% CI, 1.1 to 3.0). In survivors, patients with AKI remained on MV longer and ICU and hospital length of stay were longer than in patients without AKI. The overall mortality was 18.8% and was significantly higher for AKI patients (44.1% versus 13.3%; *P *< 0.01, OR 5.1, 95% CI, 3.3 to 7.9). Logistic regression analysis was performed with AKIN criteria, and it demonstrated that among patients with AKI, only AKI III was independently associated with higher ICU mortality (*P *< 0.001, OR 4.81, 95% CI 2.17 to 10.62).

**Conclusions:**

In our cohort of patients with H1N1 virus infection, only those cases in the AKI III category were independently associated with mortality.

## Introduction

The pandemic 2009 influenza A (H1N1) virus infection was first described in Mexico in April 2009, and several reports have been published regarding the presentation of this disease with severe acute respiratory symptoms in hospitalized patients [1]. However, the information regarding the incidence and impact of renal failure among these patients remains scarce. The World Health Organization (WHO) warned physicians that patients H1N1 virus infection might develop renal impairment ranging from just mild disease to the need for renal replacement therapy (RRT) [1-5].

In critical care settings, many studies are limited because they lack a uniform definition of acute kidney injury (AKI). The definition of AKI varies widely and is predominately based on large increments of serum creatinine kinase (CK), thus ignoring milder stages of AKI. In addition, the choice of using the Acute Kidney Injury Network (AKIN) criteria is based on the lack of reliance on baseline CK level upon intensive care unit (ICU) admission. A definition and classification of AKI were established by a consensus of critical care and nephrology societies worldwide [6]. The degree of AKI classified according to AKIN criteria correlates with mortality in a progressive fashion, emphasizing the importance of the severity of AKI. This first globally developed AKI definition and classification incorporates the important finding that small increases of serum CK levels in AKI negatively affect patient outcome.

The present study aims to evaluate whether the presence of AKI in a cohort of patients hospitalized with a severe presentation of H1N1 virus infection in the ICU is associated with worse outcomes.

## Materials and methods

Study data were obtained from a voluntary registry created by the Spanish Society of Intensive Care Medicine (SEMICYUC) after the first reported ICU case (see Additional file 1 for SEMICYUC working group investigators). Inclusion criteria were fever >38°C; respiratory symptoms consistent with cough, sore throat, myalgia or influenza-like illness; acute respiratory failure requiring ICU admission; and microbiologic confirmation of novel H1N1 virus. Data were reported by the attending physician on the basis of medical chart reviews and radiological and laboratory records. This study analyzes data from the first ICU case until 31 December 2009. Children under 15 years old were not enrolled in the study. The study was approved by the ethical board of Joan XXIII University Hospital, Tarragona, Spain. Patients remained anonymous, and the requirement for informed consent was waived because of the observational nature of the study. All tests and procedures were ordered by the attending physicians.

### Definitions

The following variables were recorded: demographic data, comorbidities, time of illness onset and hospital admission, time to first dose of antiviral delivery, microbiologic findings and chest radiologic findings at ICU admission. Intubation and mechanical ventilation (MV) requirements, adverse events during ICU stay (for example, the need for vasopressor drugs or renal replacement therapies) and laboratory findings at ICU admission were also recorded. To determine the severity of illness, the Acute Physiology and Chronic Health Evaluation II (APACHE II) score [7] was determined in all patients within 24 hours of ICU admission. Organ failure was assessed using the Sequential Organ Failure Assessment (SOFA) scoring system [8]. Obese patients were defined as those with a body mass index (BMI) over 30 kg/m^2^.

Primary viral pneumonia was defined in patients presenting illness with acute respiratory distress and unequivocal alveolar opacities involving two or more lobes with negative respiratory and blood bacterial cultures during the acute phase of influenza virus [2]. Nasopharyngeal swab specimens were collected for respiratory viruses at hospital admission, and lower respiratory secretions were also obtained from intubated patients. Real-time polymerase chain reaction (RT-PCR) testing was performed in accordance with the published guidelines from the Centers for Disease Control and Prevention (CDC) [9]. Novel influenza A H1N1 testing was performed in each institution, or centralized in a reference laboratory when not available. A confirmed case was defined as an acute respiratory illness with laboratory-confirmed pandemic H1N1 virus infection identified by RT-PCR or viral culture [10]. Only confirmed cases were included in the current study.

Community-acquired respiratory coinfection (CARC) was defined as any infection diagnosed within the first 2 days of hospitalization. Infections occurring later were considered nosocomial [11]. Patients who presented healthcare-associated pneumonia were excluded from the present study [12]. Patients were admitted to the ICU either because they were potential candidates for mechanical ventilation and/or because they were judged to be in an unstable condition requiring intensive medical or nursing care [13,14].

Oseltamivir was administered orally in accordance with CDC recommendations, and the regimen (150 mg per 24 hours or 300 mg per 24 hours) was chosen by the attending physician [15]. The ICU admission criteria and treatment decisions for all patients, including determination of the need for intubation, the dosage of RRT and the type of antibiotic and antiviral therapy administered were not standardized and were decided by the attending physician.

The AKI stages in critically ill patients with H1N1 virus infection were diagnosed according to the glomerular filtration rate criteria of the current AKIN definitions [6]. Information in regard to urine output was not used in the present manuscript. Diagnostic criteria for AKI were an abrupt (within 48 hours) reduction in kidney function, currently defined as an absolute increase in serum CK level of ≥0.3 mg/dl, a percentage increase in serum CK level of ≥50% (1.5-fold greater than baseline) or a reduction in urine output (documented oliguria of <0.5 ml/kg/hour for more than 6 hours) [6]. The severity of AKI was classified as stage I (serum CK increase of >150% to 200% (1.5- to twofold increase) or ≥0.3 mg/dl), stage II (serum CK increase of >200% to 300% (more than two- to threefold)) and stage III (serum CK increase of >300% (more than threefold) or the need for RRT). Alternatively, stage III was defined by an increase of serum CK 0.5 mg/dl from baseline serum CK values of 4.0 mg/dl. The CK criteria describe changes in renal function without specifying the direction of change. We performed an analysis of the maximum AKI severity stage reached. RRT in the course of AKI was always initiated when needed for the following indications: pulmonary edema, oliguria (defined as urine output <0.5 ml/kg body weight per hour for >6 hours), metabolic acidosis or hyperkalemia not responding to conventional treatment and uremia defined as urea nitrogen of >100 mg/dl. RRT was available 24 hours per day, and no patient requiring RRT was denied RRT on the basis of futility. All pairs of CK levels were taken within 48-hour periods and were analyzed during the course of ICU admission as the maximum AKIN stage was used.

### Statistical analysis

Discrete variables are expressed as counts (percentages) and continuous variables are expressed as means ± standard deviations (SDs) or medians with the 25th to 75th interquartile ranges (IQRs). For the demographic and clinical characteristics of the patients, differences between groups were assessed using the χ^2 ^test and Fisher's exact test for categorical variables and the Student's *t*-test or Mann-Whitney *U *test for continuous variables. Variables significantly associated with mortality in the univariate analysis were entered into the regression model. To avoid spurious associations, variables entered into the regression models were those with a relationship in univariate analysis (*P *≤ 0.05) or a plausible relationship with the dependent variable. Results are presented as odds ratios (ORs) and 95% confidence intervals (CIs). Potential explanatory variables were checked for colinearity prior to inclusion in the regression models using the tolerance and variance inflation factor. Data analysis was performed using SPSS for Windows 15.0 software (SPSS, Inc., Chicago, IL, USA).

## Results

**Figure 1:**
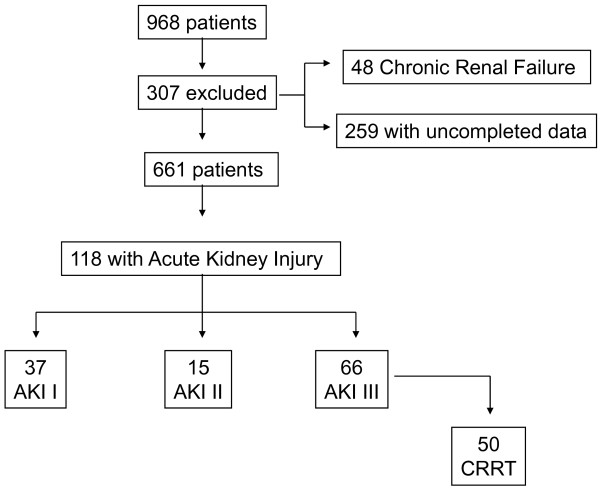
**Flowchart of critically ill patients enrolled in the study with 2009 pandemic influenza A (H1N1) virus infection**. AKI, acute kidney injury; CRRT, continuous renal replacement therapy.

A total of 968 patients from 148 Spanish ICUs were included in the database, and, after excluding patients with chronic kidney disease who were receiving dialysis treatment (*n *= 48) and patients with incomplete data (*n *= 259), a total of 661 patients were included in this study (Figure 1). Of these, 364 patients (55.1%) were male, the median age was 43 years (interquartile range (IQR, 33 to 53) and 581 patients (87.9%) were under 60 years of age. The mean APACHE II score was 13.6 ± 6.7, and the mean SOFA score was 5.4 ± 3.4 on admission. Invasive MV was used in 408 (61.7%) of the patients. All patients received antiviral therapy. Comorbidities excluding chronic renal failure were present in 466 patients (70.5%). The main comorbidities recorded were obesity (*n *= 248, 37.5%), chronic obstructive pulmonary disease (COPD; *n *= 109, 16.5%) and asthma (*n *= 87, 13.2%).

One hundred eighteen patients (17.7%) developed AKI. Patients with AKI were mostly male (65.3% versus 52.9%; *P *< 0.01) and had a mean age (±SD) of 43.8 ± 14.2 years. Patients with AKI presented comorbidities more frequently than non-AKI patients (77.1% versus 69.1%; *P *= 0.05). Patients with AKI had higher APACHE II scores (19.1 ± 8.3 versus 12.6 ± 5.9; *P *< 0.001), higher SOFA scores (8.7 ± 4.2 versus 4.8 ± 2.9; *P *< 0.001), more need of MV (87.3% versus 56.2%; *P *< 0.01, OR 5.3, 95% CI, 3.0 to 9.4), more presence of shock (75.4% versus 38.3%; *P *< 0.01, OR 4.9, 95% CI, 3.1 to 7.7), higher Multiple Organ Dysfunction Score (MODS) (92.4% versus 54.7%; *P *< 0.01, OR 10.0, 95% CI, 4.9 to 20.21) and higher CARC (23.7% versus 14.4%; *P *< 0.01, OR 1.8, 95% CI, 1.1 to 3.0) (Table 1). Patients with AKI showed higher C-reactive protein levels (median 28 mg/dl; IQR 16.8 to 61.2 versus 20 IQR 12 to 42.1; *P *< 0.01) and procalcitonin levels (median 2 ng/ml, IQR 0.8 to 10, versus 0.5 ng/ml, IQR 0.1 to 1.8; *P *< 0.01) and CK levels (median 170 U/L, IQR 74 to 417, versus 290 U/L, IQR 92.25 to 862; *P *< 0.01).

**Table 1:** Comparison of baseline characteristics for patients with or without AKI in patients affected by pandemic 2009 influenza A (H1N1) virus infection^a^

Variables	Non-AKI (*n *= 543)	AKI (*n *= 118)	Total (*n *= 661)	*P *value
Mean age, yr (±SD)	43.5 (13.9)	44.9 (15.2)	43.8 (14.2)	0.3
Male sex, *n *(%)	288 (53%)	77 (65.3%)	365 (55.2%)	0.01
Comorbidities, *n *(%)				
Pregnancy	34 (6.3%)	5 (4.3%)	39 (5.9%)	0.5
COPD	90 (16.5%)	19 (16.2%)	109 (16.5%)	0.9
Asthma	76 (14.0%)	11 (9.4%)	87 (13.2%)	0.2
Heart failure	29 (5.3%)	10 (8.5%)	39 (5.9%)	0.2
Obesity	196 (36.0%)	52 (44.4%)	248 (37.5%)	0.09
Diabetes	52 (9.6%)	19 (16.2%)	71 (10.7%)	0.04
Immunosupression	17 (3.1%)	3 (2.6%)	20 (3.0%)	0.9
Hematologic disease	26 (4.8%)	8 (6.8%)	34 (5.1%)	0.3
Neuromuscular disease	21 (3.9%)	2 (1.7%)	23 (3.5%)	0.4
HIV infection	12 (2.2%)	3 (2.6%)	15 (2.3%)	0.7

^a^AKI, acute kidney injury; COPD, chronic obstructive pulmonary disease; HIV, human immunodeficiency virus.

Thirty-seven (31.4%) of the patients with AKI were classified as AKI I, 15 (12.7%) were classified as AKI II and 66 (55.9%) were classified as AKI III, of which 50 patients (75.7%) required continuous renal replacement therapy (CRRT). Additional clinical characteristics of patients with H1N1 virus infection in accordance with AKI classifications are presented in Table 2.

**Table 2:** Selected physiologic and laboratory characteristics of patients with pandemic 2009 influenza A (H1N1) virus infection with or without AKI and AKIN criteria^a^

Variables	Total	Non-AKI (*n *= 543)	AKI (*n *= 118)	*P *value	AKI I (*n *= 37)	AKI II (*n *= 15)	AKI III (*n *= 66)	*P *value
Physiologic characteristics								
Mean APACHE II score (±SD)	13.6 (6.7)	12.6 (5.9)	19.1 (8.4)	<0.001	16.6 (6.9)	20.9 (7.4)	20.8 (9.3)	<0.001
Mean SOFA score (±SD)	5.4 (3.5)	4.8 (2.9)	8.7 (4.2)	<0.001	4.7 (2.9)	7.7 (3.5)	9.2 (4.4)	<0.001
Invasive MV, *n *(%)	408 (61.7%)	305 (56.2%)	103 (87.3%)	<0.001	28 (75.7%)	12 (80.0%)	63 (95.5%)	<0.001
Shock, *n *(%)	297 (44.9%)	208 (38.3%)	89 (75.4%)	<0.001	23 (62.2%)	8 (53.3%)	58 (87.9%)	<0.001
MODS, *n *(%)	406 (61.4%)	297 (54.7%)	109 (92.4%)	<0.001	29 (78.4%)	14 (93.3%)	66 (100.0%)	<0.001
Coinfection, *n *(%)	106 (16.0%)	78 (14.4%)	28 (23.7%)	<0.01	10 (27.0%)	5 (33.3%)	13 (19.7%)	0.03
Median laboratory findings, median (IQR)								
Leukocyte count per mm^3^	6,900 (4,000 to 11,500)	6,800 (3,925 to 11,075)	8,300 (4,300 to 14,000)	<0.01	6,770 (4,250 to 15,850)	8,850 (4,375 to 11,525)	8,200 (4,200 to 13,750)	0.5
Platelet count per mm^3^	163.5 (120 to 223.2)	166 (124 to 227)	149 (99 to 197)	0.09	160 (110 to 238)	140 (81 to 181)	149 (77.5 to 197.5)	0.02
Serum creatinine kinase, U/L	176.5 (75 to 474.2)	170 (74-417-75)	290 (92.25 to 862)	<0.01	199 (36 to 1,270)	218 (48 to 475)	319 (136.5 to 860.25)	0.005
Serum lactate dehydrogenase, IU/L	611 (366.5-1,019.7)	600 (355 to 986)	720 (402 to 1,103)	0.001	506 (305 to 954)	380 (338 to 439)	1,000 (606 to 1,527)	<0.001
Serum AST, IU/L	53 (32 to 99)	50 (31.25 to 88.75)	64 (36.5 to 147)	0.001	47 (29.5 to 111)	120 (48.5 to 204)	75 (50 to 176)	<0.001
Serum ALT, U/L	39.5 (23 to 78)	38 (23 to 76)	49.5 (26 to 96.75)	0.001	52.5 (24.75 to 83.5)	46.5 (22.5 to 89.5)	48.5 (26.5 to 129.75)	0.1
PCT, ng/ml	0.59 (0.1 to 2.1)	0.5 (0.1 to 1.8)	2 (0.8 to 10)	0.001	2 (0.57 to 5.72)	8.3 (3.7 to 10.0)	2 (0.7 to 6.9)	<0.001
CRP, mg/ml	21.1 (12.2 to 44.8)	20 (12 to 42.1)	28 (16.8 to 61.2)	<0.01	34 (16.1 to 63.7)	29 (8.6 to 44.6)	25.8 (19.2 to 69)	0.08

^a^AKI, acute kidney injury; AKIN, Acute Kidney Injury Network; APACHE II, Acute Physiology and Chronic Health II; SD, standard deviation; SOFA, sequential organ failure assessment; MV, mechanical ventilation; MODS, Multiple Organ Dysfunction Score; IQR, interquartile range; AST, aspartate aminotransferase; ALT, alanine aminotransferase; PCT, procalcitonin; CRP, C-reactive protein.

Among survivors, patients with AKI remained on MV longer (13.6 ± 15.2 versus 8.4 ± 11.5 days; *P *= 0.003), ICU length of stay (19.4 ± 16.5 days versus 12.6 ± 13.0 days; *P *< 0.0001), length of hospitalization (30.3 ± 19.9 days versus 20.5 ± 16.8 days; *P *< 0.0001) than non-AKI patients (Table 3).

**Table 3:** Outcomes of patients with pandemic 2009 influenza A (H1N1) virus infection. with or without AKI and AKIN criteria^a^

Variables	Non AKI *n *= 543	AKI *n *= 118	*P *value	AKI I *n *= 37	AKI II *n *= 15	AKI III *n *= 66	Total	*P *value
ICU death, *n *(%)	72 (13.3%)	52 (44.1%)	<0.001	9 (24.3%)	5 (33.3%)	38 (57.6%)	124 (18.8%)	<0.001
MV days^b^								
Mean (±SD)	8.4 (11.5)	13.6 (15.2)	<0.001	13.3 (17.6)	9.3 (11.8)	16.4 (12.5)	9.0 (12.0)	0.01
Median (IQR)	4 (0 to 12)	10 (3.75 to 21.5)		8 (3.25 to 20.75)	5 (0 to 14.5)	15 (5.5 to 26.5)	5 (0 to 13)	
LOS ICU^c^								
Mean (±SD)	12.6 (13)	19.4 (16.5)	<0.001	19.6 (18.4)	13.4 (11.5)	22.1 (15.3)	13.4 (13.6)	<0.001
Median (IQR)	8 (4 to 17)	13 (7 to 30)		12 (7 to 29.5)	8 (5.5 to 19.5)	21.5 (7 to 75)	9 (4 to 18)	
Hospital LOS^c^								
Mean (±SD)	20.5 (16.8)	30.3 (19.9)	<0.001	29.3 (21.4)	23.0 (14.7)	36.0 (19.0)	21.6 (17.5)	<0.001
Hospital median (IQR)	15 (9 to 27)	26.5 (13.75 to 44.25)		24.5 (13 to 44.5)	20 (10 to 34.5)	35 (19.5 to 49)	16 (9 to 29)	

^a^AKI, acute kidney injury; AKIN, Acute Kidney Injury Network; ICU, intensive care unit; MV, mechanical ventilation; IQR, interquartile range; LOS, length of stay; ICU, intensive care unit; SD, standard deviation; ^b^only survivors and mechanically ventilated; ^c^only survivors.

**Figure 2:**
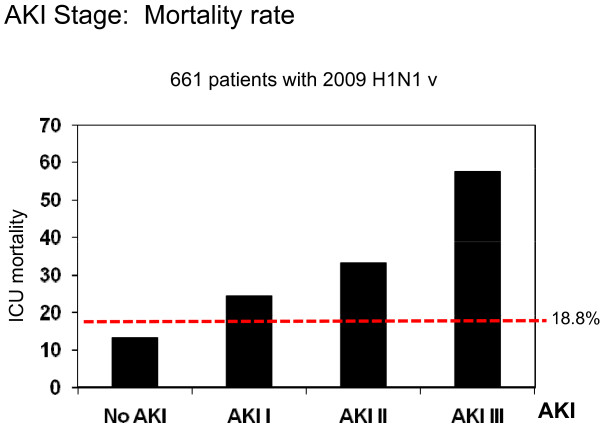
**Intensive care unit (ICU) mortality among patients with pandemic 2009 influenza A (H1N1) virus infection and Acute Kidney Injury Network (AKIN) criteria (No AKI, AKI I, AKI II, AKI III)**. Red dashed line represents the overall mortality.

Overall ICU mortality was 18.8%, and this mortality rate was significantly higher for AKI patients than for non-AKI patients (44.1% versus 13.3%; *P *< 0.01, OR 5.1, 95% CI 3.3 to 7.9). AKIN categories were based on four mutually exclusive variables. ICU mortality in patients defined by AKIN criteria was as follows: no AKI 13.3%, AKI I 24.3%, AKI II 33.3% and AKI III 57.6% (*P *< 0.0001) (Figure 2). In addition, Table 4 shows that APACHE II, SOFA, invasive MV, shock, MODS, hematologic disease and bacterial coinfection were variables associated with ICU mortality (univariate analysis). Logistic regression analysis was performed with previous significantly associated variables from the univariate analysis and with AKIN categories. Multivariate analysis demonstrated that among patients with AKI, only AKI III was independently associated with higher ICU mortality (OR 4.81, 95% CI 2.17 to 10.62; *P *< 0.001) with a Hosmer-Lemeshow goodness of fit test of 3.44 (*P *= 0.903) for the model (Table 5). In addition, with the aim of validating these results and to avoid a survival advantage of patients who died very early after ICU admission, logistic regression analysis was performed excluding patients who died within the first 48 hours in the ICU. The result of this analysis was highly consistent with the previous one (OR 5.31, 95% CI 2.37 to 11.91; *P *< 0.001).

**Table 4:** Comparison of demographic and clinical characteristics among with pandemic 2009 influenza A (H1N1) virus infection^a^

Variables	Survivors (*n *= 527)	Nonsurvivors (*n *= 134)	*P *value
Mean age, yr (±SD)	43.2 (13.9)	46.09 (15.2)	0.1
Male sex, *n *(%)	290 (54.0%)	74 (59.7%)	0.2
Mean APACHE II score (±SD)	12.5 (5.9)	18.8 (7.9)	<0.001
Mean SOFA score (±SD)	4.8 (2.8)	8.1 (4.3)	<0.001
Comorbidities, *n *(%)			
Pregnancy	33 (6.1%)	6 (4.8%)	0.6
COPD	94 (17.5%)	15 (12.1%)	0.1
Asthma	76 (14.2%)	11 (8.9%)	0.1
Heart failure	28 (5.2%)	11 (8.9%)	0.1
Obesity	194 (36.1%)	54 (43.5%)	0.1
Diabetes	56 (10.4%)	15 (12.1%)	0.6
Immunosuppression	13 (2.4%)	7 (5.6%)	0.07
Hematological disease	19 (3.5%)	15 (12.1%)	<0.001
Neuromuscular disease	17 (3.2%)	6 (4.8%)	0.4
HIV infection	11 (2.0%)	4 (3.2%)	0.4
Invasive MV	290 (54.0%)	118 (95.2%)	<0.001
Shock	208 (38.7%)	89 (71.8%)	<0.001
MODS	299 (55.7%)	107 (86.3%)	<0.001
Coinfection	76 (14.2%)	30 (24.2%)	<0.01

^a^Survivors versus nonsurvivors. APACHE II, Acute Physiology and Chronic Health Evaluation II; SOFA, sequential organ failure assessment; COPD, chronic obstructive pulmonary disease; HIV, human immunodeficiency virus; MV, mechanical ventilation; MODS, Multiple Organ Dysfunction Score.

**Table 5:** Multivariate logistic regression analysis: risk factors for ICU mortality based on AKI criteria^a^

Variables	*B*	Wald	*P *value	OR	95% CI
AKI					
AKI I	0.42	0.79	0.37	1.52	0.61 to 3.81
AKI II	0.61	0.81	0.36	1.83	0.48 to 6.90
AKI III	1.57	15.06	0.000	4.81	2.17 to 10.62
APACHE II	0.05	5.87	0.01	1.06	1.01 to 1.11
Constant	-5.19	60.58	0.00	0.00	

^a^ICU, intensive care unit; AKI, acute kidney injury; APACHE II, Acute Physiology and Chronic Health Evaluation II; OR, odds ratio; CI, confidence interval; *B*, *B *coefficient; Wald, Wald coefficient.

## Discussion

To the best of our knowledge, this is the largest study to date focusing on AKI during the H1N1 virus pandemic. The main finding of the present study was that the presence of AKI in ICU patients with a severe presentation of H1N1 virus infection was associated with increased mortality rates. In addition, only AKI III patients who were included showed higher rates and were found to have an independent risk factor for ICU mortality.

AKI is a complex disorder that occurs in a variety of settings, with clinical manifestations ranging from a minimal elevation in serum CK level to anuric renal failure. It is often underrecognized and is associated with severe consequences [16]. Renal impairment is common in ICU patients and is associated with high mortality rates and high consumption of resources, especially in patients who require RRT. Recent epidemiological studies have demonstrated the wide variation in etiologies of and risk factors for AKI [17-19]. AKI occurs in approximately 19% of patients with moderate sepsis, 23% of patients with severe sepsis and 51% of patients with septic shock [20]. Patients who have sepsis-related AKI have much higher mortality than patients with AKI who do not have sepsis [21]. Ostermann *et al. *[22] recently demonstrated that the risk of death is higher in patients with a worse degree of AKI, and only AKI III was independently associated with ICU mortality.

The mortality in AKI observed in patients with H1N1 virus has been previously reported in other forms of critical illness, particularly severe sepsis. Lopes *et al. *[23] conducted a retrospective study of a cohort of 315 patients with sepsis admitted to the infectious diseases ICU to determine the impact of AKI during ICU admission and found that AKI had a negative impact on in-hospital mortality of patients with sepsis. As compared with patients without acute renal impairment, patients with AKI had a 25.3% increased probability of death. Moreover, Lopes *et al*. found that the AKIN criteria were a useful tool to characterize and stratify septic patients according to the risk of death. In addition, the cause-and-effect relationship between viral infection and kidney injury is not clear [24]. A cause-and-effect relationship has been implied by the patients' clinical course in some studies. One possible mechanism is glomerular deposition of viral antigens, which seems to be secondary to the deposition of immune complexes. That is, the abnormal expression of cytokine dysregulation associated with severe viral infection injury might contribute to the renal injury of H1N1 virus infection. Bermejo-Martin *et al. *[25] recently reported an early secretion of Th17 and Th1 cytokines in patients with severe H1N1 virus infection. In addition, To *et al*. [26] demonstrated a slower control of viral load in patients with an exuberant cytokine. Increased cytokines, together with lymphokines, lead to the adhesion of inflammatory cells to endothelium and other injury sites [27]. Endothelium-dependent vasodilation is a prominent feature in patients with moderate renal impairment [28], and plasma cytokine levels could be useful in predicting mortality rates in critically ill patients with AKI.

H1N1 virus infection is associated with a high fatality rate [1-4]; however, a potential explanation for such rates has not been totally elucidated. Patients who require ICU admission have frequently experienced rapidly progressive, serious lower respiratory tract disease. Other well-recognized influenza complications in these seriously ill patients with H1N1 virus infection have included renal failure; however, the exact impact has not been extensively investigated. In the first case reports, impairment of renal function was commonly described, and patients who died had documented multiple organ failure with significantly higher rates of renal failure [29,30]. Myalgia is usually prominent early in the illness, in contrast to available descriptions of influenza-associated myositis, where onset occurs after or during resolution of respiratory symptoms. Although direct muscle invasion by the virus is one of the possibilities suggested for virus-related rhabdomyolysis, not all the patients who developed AKI showed high levels of CK. In addition, AKI has been reported worldwide during the last pandemic with very different incidences and a paucity of robust AKI definitions. Data from Chile reported that 25% of patients manifested elevated CK levels. Sood *et al. *[31], in a cohort of 50 critically ill patients, and Trimarchi *et al. *[32], in a study comprising 22 patients, reported an incidence around 65%. In our study, 17.7% of patients developed AKI. Differences with other studies might be related to our critically ill population, for whom the criteria were standardized on the basis of AKIN criteria. Finally, mortality rates of 16%, 19% and 54%, respectively, have been reported among critically ill patients with H1N1 virus infection in Brazil [33], Argentina [5] and Canada [3]. The main difference is that in the present study, although the mortality rate was 18.8% and significantly higher for patients who developed AKI, multivariate analysis demonstrated that only AKIN stage III was independently associated with ICU mortality.

The present study has some limitations that should be addressed. First, this is an observational, noninterventional study in which 148 ICUs were selected. Management of patients was not standardized, and management practices were chosen in accordance with local protocols. Nevertheless, the study has the strength of being a prospective, multicentered study with a large number of patients. Second, in the present study, notes were not reviewed to check for the context of patients' clinical presentations, and fluid resuscitation was not employed. In addition, the information in regard to urine output and estimated baseline CK levels was not used; this was the reason for the choice of this system based on the AKIN criteria instead of another other system of classification of AKI, such as risk, injury, failure, loss, and end-stage kidney disease (RIFLE) [34,35]. The degree of AKI classified by both the RIFLE and AKIN criteria correlates with mortality in a progressive fashion, emphasizing the importance of the severity of AKI. Both classification systems help to standardize the definition and management of AKI. In the present analysis, the AKIN criteria were chosen for analysis instead of the RIFLE criteria. The choice of AKIN criteria may have been driven by the lack of reliance on baseline CK levels, which the RIFLE criteria do not take into consideration. Also, the RIFLE criteria do not consider the nature or site of the kidney injury [36]. Finally, a potential bias might have occurred because a diagnosis of AKI as a baseline hazard ignores some patients who may have died very early, before a diagnosis of AKI could be made. To avoid this potential bias, the multivariate analysis was performed after excluding patients who died within the first 48 hours after ICU admission and after it was confirmed that AKI III was associated with a statistically significant worse outcome. In addition, as reported by other authors [21], some patients who were receiving CRRT would have been classified as having AKI I or AKI II, which might have altered their outcome. Future research seems mandatory to clarify the complexities and confounding factors of AKI.

## Conclusions

In summary, AKI represents a frequent complication in critically ill patients with H1N1 virus infection and is associated with increased mortality; however, only AKI stage III was independently associated with worse outcome. In addition, AKI was associated with increased use of healthcare resources as manifested by increased ICU and hospital LOS and more days under MV.

## Key messages

• AKI represents a frequent complication in critically ill patients with H1N1 virus infection.

• AKI development in critically ill patients with H1N1 virus infection is associated with worse outcome.

• Only critically ill patients affected by pandemic H1N1 virus infection in stage AKI III are independently associated with increased mortality.

• AKI development in critically ill patients affected by H1N1 virus infection is associated with consumption of increased health care resources manifested by increased ICU and hospital LOS and more days under mechanical ventilation.

• Prompt supportive measures are warranted in critically ill patients with H1N1 virus infection to decrease the development of AKI.

## Abbreviations

AKI: acute kidney injury; AKIN: Acute Kidney Injury Network, APACHE II: Acute Physiology and Chronic Health Evaluation II; BMI: body mass index; CAP: community-acquired pneumonia; CDC: Centers for Disease Control and Prevention; CI: confidence interval; CK: creatinine kinase; COPD: chronic obstructive pulmonary disease; CRP: C-reactive protein; CRRT: continuous renal replacement therapy; ESKD: end-stage kidney disease; HIV: human immunodeficiency virus; HR: hazard ratio; ICU: intensive care unit; IQR: interquartile range; LOS: length of stay; MODS: Multiple Organ Dysfunction Score; MV: mechanical ventilation; OR: odds ratio; PCT: procalcitonin; RIFLE: risk, injury, failure, loss, and end-stage kidney disease; RRT: renal replacement therapy; RT-PCR: real-time polymerase chain reaction; SD: standard deviation; SOFA: Sequential Organ Failure Assessment; WHO: World Health Organization.

## Competing interests

The authors declare that they have no competing interests.

## Authors' contributions

AR made a substantial contribution. AR and IML assisted in the design of the study, coordinated patient recruitment, analysed and interpreted the data and assisted in writing the paper. RZ, RG, LS, JB, MV, JCP, PL, JJN, MLC and AA made important contributions to the acquisition and analysis of data. EP and DS were involved in revising the manuscript critically for important intellectual content. JR and ED made substantial contributions to the conception, design, analysis and interpretation of data and revised the final manuscript version. All authors read and approved the final manuscript.

## Supplementary Material

Additional file 1 **H1N1 SEMICYUC Working Group investigators**. Click here for file
