# Supplementary material for: Acute kidney injury in critical ill patients affected by influenza A (H1N1) virus infection
Source: Crit Care. 2011 Feb 22;15(1):R66. doi: 10.1186/cc10046 (PMC3221999; doi:10.1186/cc10046)
Supplement: Additional file 1 — H1N1 SEMICYUC Working Group investigators. [file cc10046-S1.DOCX]

Additional file 1: H1N1 SEMICYUC Working Group investigators

**Andalucía :** Pedro Cobo (**Hospital Punta de Europa, Algeciras**); Javier Martins (**Hospital Santa Ana Motril, Granada**); Cecilia Carbayo (**Hospital Torrecardenas, Almería**);Emilio Robles-Musso, Antonio Cárdenas,Javier Fierro **(Hospital del Poniente, Almería**); Ocaña Fernández (**Hospital Huercal – Overa, Almería**); Rafael Sierra (**Hospital Puerta del Mar, Cádiz**); Mª Jesús Huertos **(Hospital Puerto Real, Cádiz**); Juan Carlos Pozo , R. Guerrero (**Hospital Reina Sofía , Córdoba**); Enrique Márquez (**Hospital Infanta Elena, Huelva**) ; Manuel Rodríguez-Carvajal (**Hospital Juan Ramón Jiménez, Huelva**); Antonio Jareño , (**Hospital del SAS de Jerez , Jerez de la Frontera**); José Pomares, José Luis Ballesteros (**Hospital Universitario San Cecilio, Granada**) ; Yolanda Fernández, Francisco Lobato, José F. Prieto, José Albofedo-Sánchez (**Hospital Costa del Sol , Marbella**); Pilar Martínez (**Hospital Vírgen de la Victoria , Málaga**) ; Miguel Angel Díaz Castellanos, (**Hospital Santa Ana de Motril, Granada**) ; Guillermo Sevilla, (**Clínica Sagrado Corazón, Sevilla**); José Garnacho-Montero , Rafael Hinojosa, Esteban Fernández , (**Hospital Virgen del Rocío , Sevilla**); Ana Loza, Cristóbal León (**Hospital Universitario Nuestra Señora de Valme, Sevilla**); Angel Arenzana,(**Hospital Virgen de la Macarena, Sevilla**), Dolores Ocaña (**Hospital de la Inmaculada, Sevilla),** Inés Navarrete **(Hospital Virgen de las Nieves, Granada)**,

**Aragón:** Manuel Luis Avellanas, Arantxa Lander, S Garrido Ramírez de Arellano, MI Marquina Lacueva (**Hospital San Jorge, Huesca**); Pilar Luque (**Hospital Lozano Blesa , Zaragoza**); Ignacio González (**Hospital Miquel Servet, Zaragoza**) ; Jose Mª Montón (**Hospital Obispo Polanco, Teruel**) ; Jose Mª Díaz, Pilar López-Reina, Sergio Sáez , (**Hospital Virgen de la Salud, Teruel**).

**Asturias:** Lisardo Iglesias, Carmen Pascual González (**Hospital Universitario Central de Asturias – HUCA, Oviedo**); Quiroga ( **Hospital De Cabueñes, Gijón**); Águeda García-Rodríguez ( **Hospital Valle del Nalón, Langreo**).

**Baleares:** Lorenzo Socias , Pedro Ibánez, Marcío Borges-Sa; A. Socias, Del Castillo A **( Hospital Son LLatzer ,Palma de Mallorca**);Ricard Jordà Marcos (**Clínica Rotger , Palma de Mallorca)**; José M Bonell (USP**. Clínica Palmaplanas, Palma de Mallorca**); Ignacio Amestarán (**Hospital Son Dureta, Palma de Mallorca**).

**Canarias:** Sergio Ruiz- Santana , Juan José Díaz,(**Hospital Dr Negrín ,Las Palmas de Gran Canaria**); Sisón (**Hospital Doctor José Molina, Lanzarote**); David Hernández , Ana Trujillo, Luis Regalado, (**Hospital General la Palma, La Palma**); Leonardo Lorente ( **Hospital Universitario de Canarias, Tenerife**) ; Mar Martín ( **Hospital de la Candelaria, Tenerife**), Sergio Martínez, J.J.Cáceres ( **Hospital Insular de Gran Canaria**).

**Cantabria:** Borja Suberviola, P. Ugarte, (**Hospital Universitario Marqués de Valdecilla, Santander**);

**Castilla La Mancha:** Fernando García-López, (**Hospital General, Albacete**); Angel Álvaro Alonso, Antonio Pasilla (**Hospital General La Mancha Centro, Alcázar de San Juan**); Mª Luisa Gómez Grande **(Hospital General de Ciudad Real, Ciudad Real**); Antonio Albaya, (**Hospital Universitario de Guadalajara, Guadalajara**); Alfonso Canabal , Luis Marina, (**Hospital Virgen de la Salud , Toledo**).

**Castilla y León:** Juan B López Messa,( **Complejo Asistencial de Palencia, Palencia**) , Mª Jesús López Pueyo (**Hospital General Yagüe, Burgos**); Zulema Ferreras, (**Hospital Universitario de Salamanca, Salamanca**); Santiago Macias, (**Hospital General de Segovia, Segovia**); José Ángel Berezo, Jesús Blanco Varela, (**Hospital Universitario Río Hortega , Valladolid**), Andaluz Ojeda A (**Hospital Universitario, Valladolid);** Antonio Álvarez Terrero (**Hospital Virgen de la Concha, Zamora),** Fabiola Tena Ezpeleta **(Hospital Santa Bárbara , Soria)**

**Cataluña:** Rosa Mª Catalán **( Hospital General de Vic, Vic**); Miquel Ferrer , Antoni Torres (**Hospital Clínic, Barcelona**); Sandra Barbadillo **(Hospital General de Catalunya – CAPIO, Barcelona**); Lluís Cabré (**Hospital de Barcelona, Barcelona**); Assumpta Rovira (**Hospital General de l'Hospitalet, L’Hospitalet**);Francisco Álvarez-Lerma, Antonia Vázquez, Joan Nolla ( **Hospital Del Mar, Barcelona**); Francisco Fernández, Joaquim Ramón Cervelló (**Centro Médico Delfos, Barcelona**); Rafael Mañéz , J. Ballús , Rosa Mª Granada(**Hospital de Bellvitge, Barcelona**); Jordi Vallés, Marta Ortíz , C. Guía ( **Hospital de Sabadell, Sabadell**); Fernando Arméstar, Joaquim Páez (**Hospital Dos De Mayo , Barcelona**); Jordi Almirall ,Xavier Balanzo (**Hospital de Mataró , Mataró**); Jordi Rello, Elena Arnau , Lluis Llopart, Mercedes Palomar (**Hospital Vall d'Hebron, Barcelona**); Iñaki Catalán (**Hospital Sant Joan de Déu , Manresa**); Josep Mª Sirvent, Cristina Ferri, Nerea López de Arbina (**Hospital Josep Trueta , Girona**); Mariona Badía, Montserrat Valverdú- Vidal, Fernando Barcenilla (**Hospital Arnau de Vilanova , Lleida**); Mònica Magret , (**Hospital Sant Joan de Reus, Reus**); MF Esteban, José Luna, (**Hospital Verge de la Cinta, Tortosa**); Juan Mª Nava , J González de Molina , (**Hospital Universitario Mutua de Terrassa , Terrassa**);Zoran Josic (**Hospital de Igualada , Igualada**); Francisco Gurri (**Hospital Quirón, Barcelona** , Alejandro Rodríguez, Thiago Lisboa, Diego de Mendoza, Sandra Trefler (**Hospital Universitario Joan XXIII, Tarragona),** Rosa María Díaz (**Hospital San Camil . Sant Pere de Ribes, Barcelona)**

**Extremadura:** Juliá-Narváez José (**Hospital Infanta Cristina, Badajóz**), Alberto Fernández-Zapata, Teresa Recio, Abilio Arrascaeta , Mª José García-Ramos , Elena Gallego (**Hospital San Pedro de Alcántara, Cáceres**); F. Bueno (**Hospital Virgen del Puerto, Plasencia)**.

**Galicia:** Mª Lourdes Cordero, José A. Pastor, Luis Álvarez – Rocha (**CHUAC, A Coruña**); Dolores Vila, (**Hospital Do Meixoeiro , Vigo**) ; Ana Díaz Lamas (**Hospital Arquitecto Marcide, Ferrol**); Javier Blanco Pérez, M Ortiz Piquer , (**Hospital Xeral - Calde, Lugo**); Eleuterio Merayo, Victor Jose López-Ciudad, Juan Cortez, Eva Vilaboy (**Complejo Hospitalario de Ourense , Ourense**) ; Eva Maria Saborido, (**Hospital Montecelo, Pontevedra**); Raul José González, (**H. Miguel Domínguez, Pontevedra**); Santiago Freita , (**Complejo Hospitalario de Pontevedra , Pontevedra**).

**La Rioja:** José Luis Monzón, Félix Goñi (**Hospital San Pedro, Logroño)**.

**Madrid:** Frutos Del Nogal Sáez, M Blasco Navalpotro (**Hospital Severo Ochoa, Madrid**); Mª Carmen García-Torrejón, (**Hospital Infanta Elena , Madrid**) ;César Pérez –Calvo, Diego López( **Fundación Jiménez Díaz, Madrid**); Luis Arnaiz, S.Sánchez- Alonso, Carlos Velayos, (**Hospital Fuenlabrada , Madrid**) ; Francisco del Río, Miguel Ángel González **(Hospital Clínico San Carlos, Madrid**) ; María Cruz Martín , José Mª Molina (**Hospital Nuestra Señora de América, Madrid**); Juan Carlos Montejo , Mercedes Catalán ( **Hospital Universitario 12 de Octubre, Madrid**); Patricia Albert, Ana de Pablo ( **Hospital del Sureste, Arganda del rey** ) ;José Eugenio Guerrero,  Jaime Benitez Peyrat (**Hospital Gregorio Marañón, Madrid**); Enrique Cerdá, Manuel Alvarez, Carlos Pey, ( **Hospital Infanta Cristina, Madrid**);Montse Rodríguez, Eduardo Palencia (**Hospital Infanta Leonor, Madrid**); Rafael Caballero,( **Hospital de San Rafael, Madrid**); Rafael Guerrero (**Hospital Reina Sofía, Madrid**); Concepción Vaquero , Francisco Mariscal, S. García, (**Hospital Infanta Sofía, Madrid**);Almudena Simón (**Hospital Nuestra Señora del Prado, Madrid**); Nieves Carrasco, (**Hospital Universitario La Princesa, Madrid**); Isidro Prieto, A Liétor, R. Ramos (**Hospital Ramón y Cajal, Madrid**);Beatríz Galván, Juan C. Figueira, M. Cruz Soriano (**Hospital La Paz, Madrid**) ; P Galdós; Bárbara Balandin Moreno (**Hospital Puerta de Hierro, Madrid**); Fernández del Cabo (**Hospital Monte Príncipe, Madrid**); Cecilia Hermosa, Federico Gordo ( **Hospital de Henares, Madrid);**  Alejandro Algora (**Hospital Universitario Fundación Alcorcón, Madrid**); Amparo Paredes( **Hospital Sur de Alcorcón, Madrid**); JA Cambronero (**Hospital Universitario Príncipe de Asturias , Madrid**); Sonia Gómez-Rosado, (**Hospital de Móstoles, Madrid**).

**Murcia:**

Sofía Martínez (**Hospital Santa María del Rosell, Murcia**); F. Felices Abad, (**Hospital Universitario Reina Sofía, Murcia)** ;Mariano Martínez, (**Hospital Universitario Virgen de la Arrixaca, Murcia**) ; Sergio Manuel Butí , Gil Rueda, Francisco García( **Hospital Morales Messeguer, Murcia).**

**Navarra:** Laura Macaya, Enrique Maraví-Poma, I Jimenez Urra , L Macaya Redin , A Tellería (**Hospital Virgen del Camino, Pamplona**); Josu Insansti , (**Hospital de Navarra, Pamplona**).

**País Vasco:** Nagore González , Pilar Marco, Loreto Vidaur ( **Hospital de Donostia, San Sebastián**); B. Santamaría,( **Hospital de Basurto, Bilbao**) ; Juan Carlos Vergara, Jose Ramon Iruretagoyena Amiano, (**Hospital de Cruces, Bilbao**) ; Alberto Manzano, (**Hospital Santiago Apóstol , Vitoria**);Carlos Castillo Arenal (**Hospital Txagorritxu, Vitoria)**.

**Valencia:** José Blanquer (**Hospital Clinic Universitari, Valencia**); Roberto Reig Valero , A. Belenger, Susana Altaba (**Hospital General de Castellón, Castellón**); Bernabé Álvarez -Sánchez , (**Hospital General de Alicante, Alicante**); Santiago Alberto Picos , ( **Hospital Torrevieja Salud, Alicante**); Ángel Sánchez-Miralles, ( **Hospital San Juan, Alicante**) ; Juan Bonastre , M . Palamo, Javier Cebrian, José Cuñat ( **Hospital La Fe , Valencia**) ; Belén Romero (**Hospital de Manises, Valencia**); Rafael Zaragoza, (**Hospital Dr Peset, Valencia**) ; Virgilio Paricio, (**Hospital de Requena , Valencia**); Asunción Marques, S. Sánchez-Morcillo, S. Tormo (**Hospital de la Ribera, Valencia**). J. Latour (**H.G Universitario de** **Elche, Valencia)**, M Ángel García (**Hospital de Sagunto, Castellón)**.
